# Discovery of putative long non-coding RNAs expressed in the eyes of *Astyanax mexicanus* (Actinopterygii: Characidae)

**DOI:** 10.1038/s41598-023-34198-5

**Published:** 2023-07-25

**Authors:** Iuri Batista da Silva, David Aciole Barbosa, Karine Frehner Kavalco, Luiz R. Nunes, Rubens Pasa, Fabiano B. Menegidio

**Affiliations:** 1 Institute of Biological Sciences, Federal University of Minas Gerais, Belo Horizonte, MG 31270-901 Brazil; 2 Laboratory of Ecological and Evolutionary Genetics, Institute of Biological and Health Sciences, Federal University of Viçosa Campus Rio Paranaíba, Rio Paranaíba, MG 38810-000 Brazil; 3 Integrated Biotechnology Center, University of Mogi das Cruzes (UMC), Av. Dr. Cândido X. de Almeida and Souza, 200 - Centro Cívico, Mogi das Cruzes, SP 08780-911 Brazil; 4 Center for Natural and Human Sciences, Federal University of ABC, São Bernardo do Campo, SP 09606-045 Brazil

**Keywords:** Long non-coding RNAs, Transcriptomics

## Abstract

*Astyanax mexicanus* is a well-known model species, that has two morphotypes, cavefish, from subterranean rivers and surface fish, from surface rivers. They are morphologically distinct due to many troglomorphic traits in the cavefish, such as the absence of eyes. Most studies on *A*. *mexicanus* are focused on eye development and protein-coding genes involved in the process. However, lncRNAs did not get the same attention and very little is known about them. This study aimed to fill this knowledge gap, identifying, describing, classifying, and annotating lncRNAs expressed in the embryo’s eye tissue of cavefish and surface fish. To do so, we constructed a concise workflow to assemble and evaluate transcriptomes, annotate protein-coding genes, ncRNAs families, predict the coding potential, identify putative lncRNAs, map them and predict interactions. This approach resulted in the identification of 33,069 and 19,493 putative lncRNAs respectively mapped in cavefish and surface fish. Thousands of these lncRNAs were annotated and identified as conserved in human and several species of fish. Hundreds of them were validated in silico, through ESTs. We identified lncRNAs associated with genes related to eye development. This is the case of a few lncRNAs associated with *sox2*, which we suggest being isomorphs of the *SOX2-OT*, a lncRNA that can regulate the expression of *sox2*. This work is one of the first studies to focus on the description of lncRNAs in *A*. *mexicanus*, highlighting several lncRNA targets and opening an important precedent for future studies focusing on lncRNAs expressed in *A*. *mexicanus*.

## Introduction

*Astyanax mexicanus* is a well-known model species in the study of the evolution of multiple traits, rapid phenotypic evolution and development of troglomorphic traits^1–4^. *A*. *mexicanus* has multiple populations distributed across surface rivers and subterranean rivers in Mexico. The surface populations, referred to as surface fish (SF) from now on, are found in rivers from the northwest of Mexico to the south of Texas, in the USA^5–7^. Populations from subterranean rivers, referred to as cavefish (CF), are found in the Mexican caves of Sierra de Guatemala, Sierra de Colmena and Sierra de El Abra, where more than 30 different populations are known to exist^5–9^. Aside from distribution, surface fishes and cavefishes are distinguishable by morphology, due to several troglomorphic traits found in cavefish populations. Cavefishes have craniofacial modifications, more and bigger neuromasts, more tastebuds, and reduction or absence of eyes and pigmentation^6–8,10–15^. Alongside morphological changes, cavefishes have also undergone changes in behavior^11,12,16–18^, circadian rhythm^19,20^, sleep^21,22^ and metabolism^23–25^. The degree of those troglomorphic traits varies among cavefish populations and is possible to find populations with an intermediate morphotype and others with an extreme morphotype, such as the Pachón cave population^6,26^.

Among those many traits, the absence of eyes has received distinguished attention in many studies, with eye development being a recurring topic in studies involving *A*. *mexicanus*. As such, we now know that the absence of eyes occurs due to a degeneration process during the initial stages of development. Until 20 h post-fertilization (hpf), the eye development in cavefish and surface fish is quite similar, however, after 40 hpf, the cavefish lens enters apoptosis, leading to a progressive degeneration process that results in absence of eyes in the adult phase^27–34^. This process was addressed under different approaches, including studies in retinal morphology and development^31,34–37^, lens defects and transplants^31,33,38,39^, quantitative trait loci analysis (QLTs)^28,40,41^, genomics^34,42–45^ and gene expression and transcriptomics^31,34,35,46–52^. As such, many genes are suggested to have a relevant role in eye development and degeneration, that includes, but not restricted to, the crystallin genes *αA-crys, cryaa, crybb1, crybb1c and crybgx*^31,52,53^, transcription factor *sox2*^53^, retinal homeobox *rx3*^42,45^, cone-rod homeobox *crx*^50,52^, *cbsa*^34^ and *dusp26*^45^. However, despite being well-studied, the eye development in *A*. *mexicanus* it’s not entirely understood, and many questions remain to be answered. For instance, non-coding RNAs have not properly been addressed in *A*. *mexicanus* apart from annotations in the genomes available. It’s unknown what role they may play in the development of troglomorphic traits, such as in the absence of eyes.

Non-coding RNAs represent more than 98% of the eukaryote’s genomes and correspond to transcripts that do not codify proteins^54,55^. Although they can be classified under different criteria, the ncRNA length is usually the most common. Under this criterion, if a ncRNA has less than 200 nucleotides (nt), it is classified as a small non-coding RNA (sncRNA), if has 200 nt or more, it’s a long non-coding RNA (lncRNA)^56,57^. Alongside this initial classification, each category has different classes. SncRNAs are organized into at least 5 classes: microRNA (miRNA), small nuclear RNA (snRNA), small nucleolar RNA (snoRNA), small interfering RNA (siRNA) and PIWI-interacting small RNA (piRNA)^57–59^. MiRNAs, for example, are essential in almost every developmental process and the disruption of miRNA genes can result in developmental defects, including retinal degeneration^60^.

On the other hand, lncRNAs are usually classified according to the genomic position, as seen in the GENCODE, in which a lncRNA can be sense, antisense, intronic and intergenic (lincRNA)^61–63^. For lncRNA transcripts, this classification is extended and considers the position, localization, and direction of transcription regarding the nearest protein-coding gene^61,64,65^. LncRNAs have many functions but are well-known to act as regulators of gene expression, acting during transcription, post-transcription and even at an epigenetic level^66–71^ As such, they are present in a variety of processes, including cellular differentiation^72^, embryonic development^73,74^ and adaptation^75^. Some lncRNAs are known to be involved in ocular diseases, including corneal neovascularization, glaucoma, cataract, and diabetic retinopathy^76–78^. The ANRIL lncRNA (antisense noncoding RNA in the INK4 locus), for example, is suggested to have a role in modulating optic nerve degeneration^79^. Hence, lncRNAs may play a role in eye degeneration in *A*. *mexicanus* and must be investigated. To do so, a broad identification and description of lncRNAs are needed.

Therefore, due to the absence of studies describing lncRNAs in *A*. *mexicanus*, we aimed to fill this knowledge gap by investigating lncRNAs expressed in the eye tissue of *A*. *mexicanus* embryos from cave and surface fish. We successfully identified thousands of putative lncRNAs expressed in both morphotypes and exclusive to each of them. Additionally, we were able to associate lncRNAs to protein-coding genes previously described as candidates in the eye development and degeneration in *A*. *mexicanus*.

## Materials and methods

### Library acquisition and pre-processing

The eye tissue RNA-Seq libraries of *A*. *mexicanus* used in this work were generated by Gore et al.^52^ and are available at the Sequence Read Archive (SRA), under the bioproject PRJNA429434, and at the Gene Expression Omnibus (GEO), access number GSE109006. Gore et al.^52^ extracted the eyes of *A*. *mexicanus* embryos at 54 h post-fertilization from each morphotype and isolated the total RNA with ZR-Duet DNA/RNA MiniPrep Kit (Zymo Research). Next, they prepared the libraries with the TruSeq RNA (Illumina) kit and enriched it for poly(a) RNA. Two biological replicates from each morphotype were sequenced in Illumina HiSeq 2500 platform (paired-end libraries, 2 × 100 bp and 100 million reads). The cavefish and surface fish libraries were deposited by the authors in SRA, under the accession numbers SRR6456919/SRR6456920 and SRR6456921/SRR6456922, respectively.

**Figure 1:**
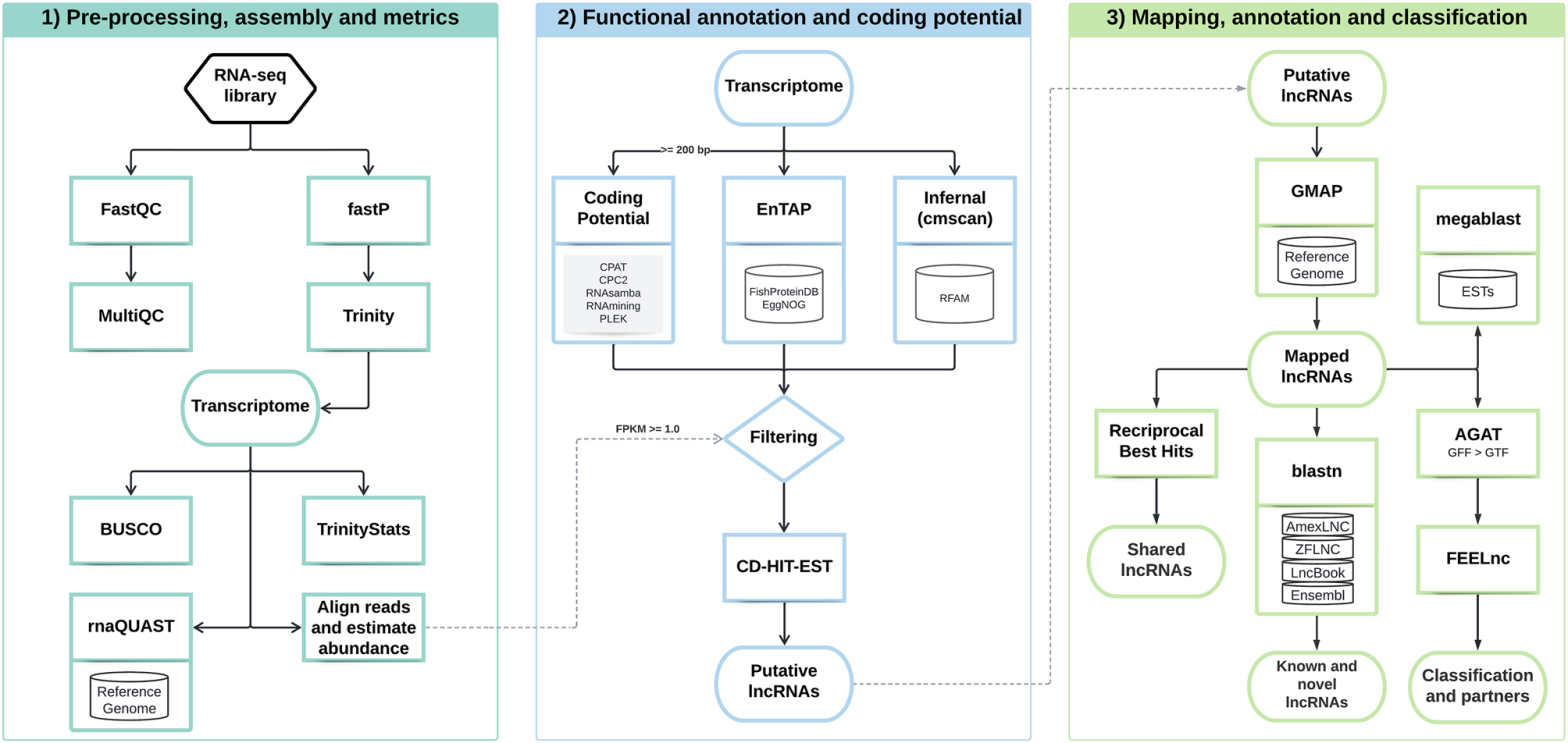
Simplified workflow of the conducted analysis, divided into three major steps. (1) Pre-processing of RNA-seq libraries, transcriptome assembly, quality assessment and general metrics; (2) Functional annotation of the transcriptomes, coding potential calculation, identification of ncRNAs families and filtering process. (3) Mapping of putative lncRNAs, identification of shared, novel, known and conserved lncRNAs, as well their classification, interactions and in silico validation of them through ESTs.

We downloaded the libraries from SRA and assessed the quality with FastQC v.0.11.9^80^ and summarized on MultiQC v.1.11^81^. Adapters, contaminants, and low-quality reads were removed with fastP v.0.20.1^82^, considering a high-quality score (> Q30). The methodology workflow, including the next steps, is summarized in Fig. 1 and detailed information on each tool, such as versions and options used, is available in Supplementary Table ST1-S1.

### De novo assembly and metrics assessment

Using the trimmed reads, we assembled eye-specific transcriptomes for both cave and surface fish through a de novo approach, using Trinity v.2.9.1^83^, integrated into Galaxy Europe webserver (https://usegalaxy.eu/). For the next steps, we removed the ‘TRINITY’ prefix in the sequence name to simplify the IDs. Assembly metrics were assessed through the TrinityStats script and FPKM values for each transcript were obtained with *align reads and estimate abundance* script, both available with the Trinity package. Transcriptome completeness was evaluated with the aid of the Benchmarking Universal Single-Copy Orthologs (BUSCO) tool v.5.0.0^84^, using the Actinopterygii OrthologDB v.10^85^ that consists of 3,640 BUSCO groups. The overall assembly quality was accessed with rnaQUAST v.2.2.1^86^, mapping the transcriptomes against the reference genomes of *A*. *mexicanus*. The cavefish genome from Pachón cave^87^ was used as a reference for the cavefish transcriptome, and the surface genome^45^ for the surface fish transcriptome.

### Functional and ncRNA annotation

The assembled transcriptomes were annotated through the Eukaryotic Non-Model Transcriptome Annotation Pipeline v.5.0.0 (EnTAP)^88^ in two steps: (a) similarity search using blastx with e-value ≤ e-5 and ≥ 50% minimum coverage against the custom database FishProteinDB (this study) and EggNOG database^89^. The FishProteinDB consists of 171,502 protein sequences of Hyperoartia, Myxini, Chondrichthyes, Actinopterygii and Sarcopterygii species (excluding the Tetrapod clade) from RefSeq. Additionally, we included proteins of *A*. *mexicanus* available in the Ensembl database; (b) functional annotation against the EggNOG databases to identify and assign Gene Ontology^90^, KEGG terms^91–93^ and protein domains from SMART^94^ and PFAM^95^.

The transcriptomes were also annotated with the *cmscan* program, part of the Infernal suite v.1.1.4^96^, using the Rfam v.14.6 database^97^ to classify transcripts into different non-coding RNA families. This step considered only annotations filtered by the bit score gathering threshold determined in Rfam.

### Long non-coding RNA prediction

To identify long non-coding RNAs, we removed transcripts with less than 200 bp from the transcriptomes. The remaining sequences were submitted to five coding potential calculator tools (CP tools): Coding Potential Calculator 2 (CPC2) py3 v.1.0.1^98^, RNASamba v.0.2.5^99^, Coding-Potential Assessment Tool (CPAT) v.3.0.4^100^, RNAmining v.1.0.4^101^ and PLEK v.1.2^102^. A similar approach was conducted by Mishra and Wang^103^ with zebrafish, using six different CP tools, and by Aciole Barbosa et al.^104^ with cobia (*Rachycentron canadum*), using three CP tools alongside EnTAP and Infernal annotations. Both works successfully identified thousands of lncRNAs, however, they did not consider misclassifications and discordant results by the CP tools, only lncRNAs predicted as non-coding by all tools. As each CP tool classified the transcripts as coding or non-coding, to avoid tool bias and misclassifications, we only considered a transcript as coding or non-coding if four out of five tools agreed with this classification. Transcripts with only 3 tools concurring on the classification were considered ambiguous and deemed undetermined.

Next, we filtered out coding and undetermined elements, as well as any sequence classified as non-coding that were annotated in the EnTAP and Infernal steps. Redundant sequences were removed with CD-HIT-EST v.4.6^105,106^, using a similarity threshold of 1.0 and a word size of 8. Transcripts with FPKM value < 1 were then removed, and the resulting transcripts were deemed as long non-coding RNA candidates. In summary, we delimited lncRNA candidates in this study as transcripts with 200 or more nucleotides, that were classified as non-coding by at least 4 coding potential tools, that were not annotated by EnTAP nor Infernal and had FPKM value ≥ 1.

### Mapping and classification of lncRNAs

LncRNA candidates were mapped against the chromosomes of their respective morphotypes with GMAP v.2021-12-17^107^, using the same genomes used in the rnaQUAST step. An index was created for each genome, excluding unplaced scaffolds and sequences not assigned to a chromosome. Chimeric alignments were then excluded from the gff3 output. Next, using the *agat_convert_sp_gff2gtf* script, available with the AGAT package v.0.9.1^108^, the GFF3 file was converted into a GTF file and used as input in FEELnc v.0.2^64^ along with the lncRNA candidates. Next, the classifier module from FEELnc was used to predict interactions between lncRNAs and mRNAs and to classify lncRNAs based on genomic position considering four categories: type, subtype, transcription direction and localization, totalizing 16 possible transcript classifications. More info about this classification is available on the FEELnc GitHub page (github.com/tderrien/FEELnc#3–feelnc_classifierpl).

Next, we summarized this lncRNA transcript classification based on the GENCODE^61^ classification, separating lncRNAs in genic and intergenic. Genic lncRNAs were organized in exonic, intronic and overlapping, and intergenic in same-strand, convergent and divergent. For the interactions between lncRNAs and partners, we filtered the results using isBest = 1, as such, only the best interactions were retained.

### Annotation of lncRNAs

To identify conserved lncRNAs between cave and surface morphotypes, we conducted a Reciprocal Best Hits (RBH) blast analysis^109,110^, also in the Galaxy Europe, with lncRNA candidates using megablast task, with identity ≥ 70% and coverage ≥ 70%. In silico validation was executed through a primary sequence alignment of the mapped lncRNA candidates against a database of Expressed Sequence Tags (ESTs) of *A*. *mexicanus*. Initially, 189,864 ESTs of *A*. *mexicanus* were downloaded from NCBI’s GenBank (https://www.ncbi.nlm.nih.gov/genbank/). We removed redundant sequences with CD-HIT-EST with the same parameters as described before, resulting in a database of ESTs with 148,690 unique ESTs. Next, we conducted the alignment with megablast task with NCBI BLAST + ^109,110^ integrated into the Galaxy Europe webserver. We applied a cutoff of e-value ≤ e−5, and identity and coverage ≥ 95%.

For the annotation step, we constructed a database of lncRNAs (AmexLNC DB), which comprehends previously described lncRNAs predicted in the previously cited genomes of *A*. *mexicanus* Pachón cave and surface, totalizing 7773 sequences. We conducted a similarity search with NCBI BLAST + under the megablast task, using the lncRNA candidates of each morphotype as query sequences, against the AmexLNC DB. An e-value ≤ e−5 was used as a cutoff, alongside identity ≥ 70% and coverage ≥ 70%. The blast results were filtered with BLAST top hit descriptions v.0.1.1^111^ and only the 3 top hits were considered.

Next, we proceed with the annotation step with two other lncRNAs databases: the Zebrafish LncRNA Database (ZFLNC)^112^, which comprehends 21,128 sequences of *Danio rerio*, and the LncBook 2.0^113^, a curated database with 323,950 transcripts sequences of human lncRNAs. While applying the same methodology used in the AmexLNC DB step, we keep the e-value ≤ e-5 as the cutoff, however, we used specific values of identity and coverage based on phylogenetic relationship. As such, for the annotation against zebrafish sequences, we used identity and coverage of 50%, while against human sequences, we maintained the identity as 50% but lowered the coverage to 25%. The choice of identity and coverage values was done considering the low sequence conservation of lncRNAs^114,115^. Venn diagrams were drawn with InteractiVenn^116^ online tool, to show lncRNAs that were annotated only against a single database and that had annotations in more than one.

### LncRNAs conservation among fishes

Initially, ncRNA sequence data from Ensembl were downloaded from Ensembl ftp (https://ftp.ensembl.org/pub/current_fasta/) for all 90 assemblies available for the group ‘Fish’ (Supplementary Table ST1-S2) to create a blast database. In a first step, the multifasta from each fish was utilized to produce a blast database and all mapped CF and SF lncRNAs were blasted against them producing xml and tabular outputs. This step allowed filtering out all fish databases in which no hits were found (e-value >  = e−3 and query coverage >  = 60%). The remaining databases were merged in a single blast database and CF/SF lncRNAs were blasted (e-value >  = e−3) in a second round. This second step allowed us to easily filter the tabular outputs (query coverage >  = 60%) to obtain lncRNAs matching fish species and visualize these alignments with the aid of BlastViewer v. 5.5.2 (https://github.com/pgdurand/BlastViewer) using the xml blast outputs. Additionally, the number of hits in each species was summarized in a dendrogram with a custom R script based on taxize^117^, myTAI^118^ and ggtree^119^ packages.

### Interactions with candidate genes for eye loss

Based on the previous data compiled by Casane and Retáux^120^ and Warren et al.^45^, we put together a list of protein-coding genes related to eye loss in the cavefish based on multiple works, considering differentially expressed genes between cavefish and surface fish, genes in QTLs related to eye development and candidates with a possible role in eye loss. We only kept the genes annotated in the *A*. *mexicanus* genomes available at Ensembl (Supplementary Table ST1-S3). We then searched for mapped lncRNAs that had at least one of these genes as a partner and created interaction plots between lncRNAs and the candidate genes using an in-house R script and the qgraph^121^ package. To enable better visualization, we removed the ‘DN’ prefix of the lncRNAs IDs in the interaction networks.

### Conservation of SOX2-OT transcripts in cavefish and surface fish

To verify if the SOX2-OT transcripts were shared between CF and SF, we filtered the RBH results previously obtained. The secondary structure of the RBH pairs and comparisons between the structure of these pairs were obtained by submitting the fasta sequences to ExpaRNA^122,123^, using default parameters.

## Results

### Transcriptome assembly and completeness assessment

The CF libraries comprised 178,682,000 (SRR6456919) and 152,354,892 (SRR6456920) raw reads. After trimming, the samples had a similar percentage of high-quality reads 174,731,916 (97.8%) and 148,191,644 (97.3%), respectively. With the trimmed reads, we successfully assembled 270,293 transcripts, comprehending 277,979,592 bases, with 43.89% GC content. As for the SF morphotype, the libraries comprised 185,809,258 (SRR6456921) and 197,626,438 (SRR6456922) raw reads, which resulted in 181,220,752 and 192,198,568 high-quality reads, after the trimming. These were assembled into 244,721 transcripts, comprising 242,667,658 assembled bases, with 44.43% GC content. These results and more detailed information are available in Supplementary Table ST1-S4.

The BUSCO completeness assessment of CF and SF transcriptomes displayed 78.0% (2840) and 80.0% (2911) of the complete BUSCO dataset (3640 elements), respectively. While the BUSCO completeness analyses provided very similar results for both CF and SF transcriptomes, they displayed significantly different proportions of single copy (27.1% and 50.3%) and duplicated BUSCOs (50.9% and 29.7%) (Supplementary Table ST2-S0 and ST3-S0). Within the *align reads and estimate abundance* results, we identified 127,764 transcripts in CF and 123,415 in SF with FPKM < 1.0 (Supplementary Table ST2–S1 and ST3–S1).

The quality assessment of assemblies with rnaQUAST revealed that almost all transcripts in CF (99.88%) and SF (98.54) had at least one significant alignment (Table 1). The number of transcripts with multiple alignments was 1.28% in CF and 0.96 in SF. Considering unaligned elements, transcripts that didn’t have a significant alignment, only 319 (0.12%) were found in CF; while in SF, unaligned transcripts were more numerous and 3574 (1.46%) transcripts were identified. The number of misassembles was similar between morphotypes, 4.86% in CF and 6.34% in SF.

**Table 1:** Results of rnaQUAST analysis for both CF and SF.

	Cavefish		Surface fish	
	Count	%	Count	%
Transcripts	270,293	100%	244,721	100%
Transcripts > 500 bp	130,902	48.43	111,470	45.55
Transcripts > 1000 bp	79,863	29.55	66,889	27.33
Aligned	269,974	99.88	241,147	98.54
Uniquely aligned	253,388	93.75	223,275	91.24
Multiply aligned	3,453	1.28	2,357	0.96
Unaligned	319	0.12	3,574	1.46
Misassemblies	13,133	4.86	15,515	6.34
Avg. aligned fraction	0.974		0.964	
Avg. alignment length	980.701		934.444	
Avg. mismatches per transcript	4.905		4.754	

Most assembled transcripts were longer than 500 bp. In CF, 99.88% of transcripts were successfully aligned against the reference genome. Similarly, in SF 98.54% were aligned. Uniquely alignments represent more than 90% of the aligned transcripts in both morphotypes.

### Functional annotation

The transcriptome annotation resulted in 105,850 (39.2%) CF and 96,100 (39.3%) SF transcripts annotated against the FishProteinDB (Supplementary Table ST2-S2 and ST3-S2). As expected, most hits in both transcriptomes represent proteins from *A*. *mexicanus*. Concerning protein domains, 93,052 (34.4%) and 83,772 (34.2%) of the transcripts of CF and SF were annotated, respectively. Looking at Gene Ontology (GO) terms, 65,711 (24.3%) transcripts of the cave were associated with biological processes, 64,798 (24.0%) with molecular function and 49,158 with cellular component terms. In the surface transcriptome, 59,550 transcripts (24.3%) were distributed into biological process terms, while 58,979 (24.1%) and 44,097 (18.0%) hit molecular functional and cellular component terms, respectively. Additionally, 29,684 (11.0%) cave and 26,763 (10.9%) surface transcripts were related to KEGG pathway terms.

### Annotated ncRNA families

**Figure 2:**
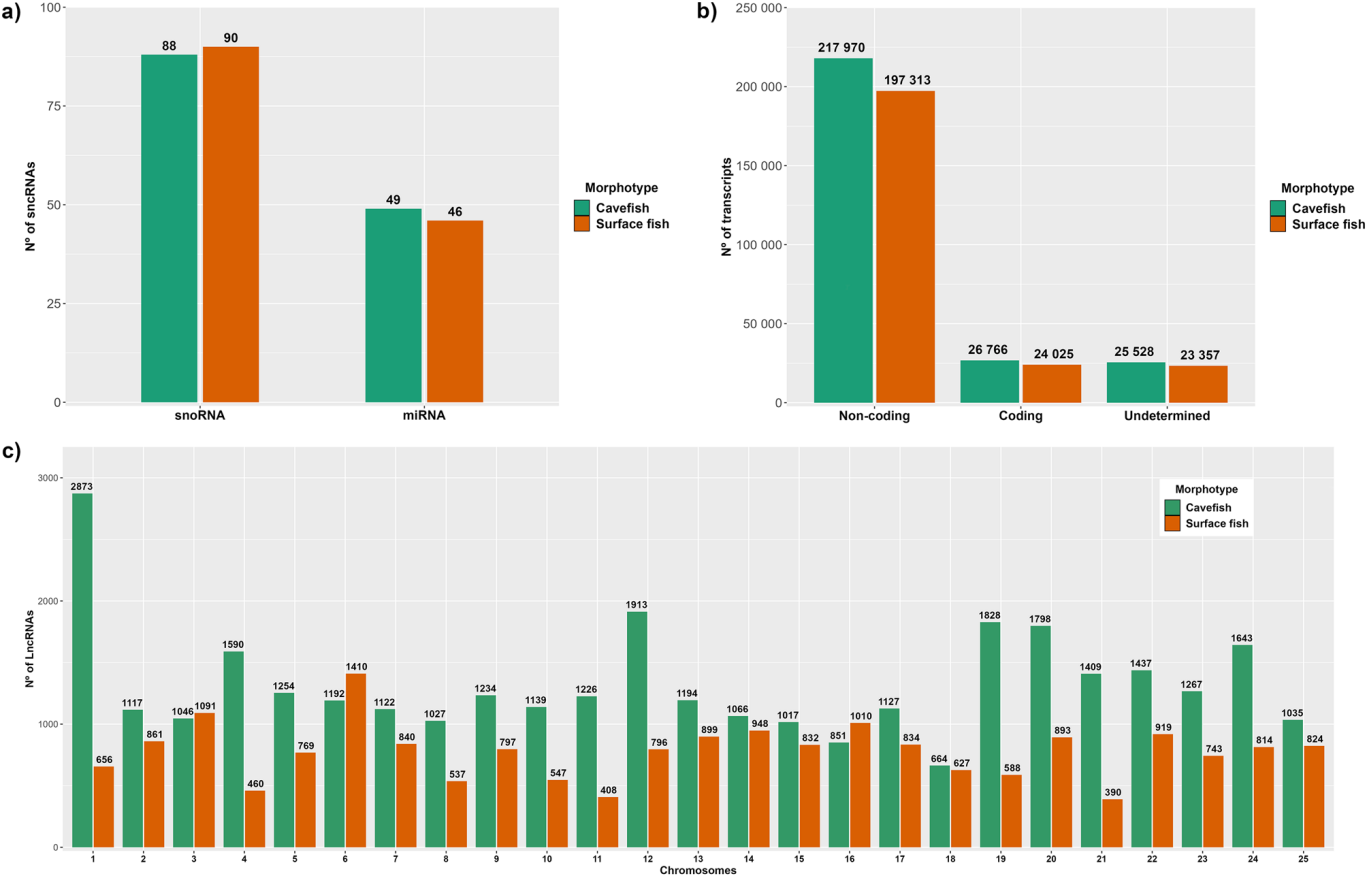
Number of sncRNAs, non-coding transcripts and the distribution of lncRNAs per chromosome. (**a**) Distribution of miRNAs and snoRNAs annotated in INFERNAL’s cmscan module; (**b**) Classification of transcripts according to their coding potential, considering the agreement of at least 4/5 coding potential tools; (**c**) Comparison of the chromosomal distribution of mapped lncRNAs between cavefish and surface fish.

Through Infernal annotation, 739 CF transcripts were found displaying similarity to 174 previously known RNAs from the Rfam database. From this total, 137 were identified as small non-coding RNAs (sncRNA), of which 88 represent small nucleolar RNAs (snoRNAs) and 49 microRNAs (miRNAs) (Fig. 2a and Supplementary Table ST2-S3). In the SF transcriptome, 699 transcripts were annotated against 173 known RNAs, with 136 being sncRNAs (90 snoRNAs and 46 miRNAs) (Fig. 2a and Supplementary Table ST3-S3).

### Long non-coding RNA candidates

The annotation of each tool to each transcript is available in Supplementary Table ST2-S4 and ST3-S4. A Venn diagram showing the agreements and disagreements between the CP tools classifications can be found in Supplementary File 1. In the analysis considering the concordance of 4/5 tools, were identified 217,970 (80.65%) non-coding and 26,766 (9.90%) coding transcripts from the CF transcriptome, while 25,528 (9.45%) elements were considered undetermined (Fig. 2b). Similarly, 197,313 (80.64%) non-coding and 24,025 (9.82%) coding transcripts from the SF transcriptome were identified, leaving 23,357 (9.54%) elements classified as undetermined (Fig. 2b).

All non-coding RNAs were filtered against EnTAP annotations, providing identification for 55,933 (CF) and 50,794 (SF) transcripts. A similar analysis was conducted against Infernal, further identifying 357 elements in CF and 325 in SF. We also removed 824 CF and 326 SF redundant sequences with CD-HIT and excluded 127,764 transcripts in CF and 123,415 in SF with FPKM < 1.0. After this filtration process, we identified 33,092 and 22,453 transcripts as lncRNA candidates, from CF and SF transcriptomes, respectively.

### Mapped lncRNAs

Considering the putative lncRNAs from CF, 33,069 (99;93%) of them were successfully mapped against the Pachón cave genome. From this totality, 2873 (8.69%) were mapped exclusively against chromosome 1, while chromosome 18 had the lowest number with only 664 (2.00%) mapped lncRNAs (Supplementary Table ST2-S5). In SF, the proportion of mapped lncRNAs was lower, with only 19,493 lncRNAs in total (86.82%) being mapped against the surface genome. Different from the scenario observed in the cavefish, only 656 (3.29%) lncRNAs were mapped against chromosome 1 and chromosome 21 had only 390 (1.96%) mapped lncRNAs, the lowest number among the 25 surface fish chromosomes (Supplementary Table ST3-S5). These differences in the number of mapped lncRNAs are present in almost all chromosomes (Fig. 2c).

### Shared and validated lncRNAs

**Figure 3:**
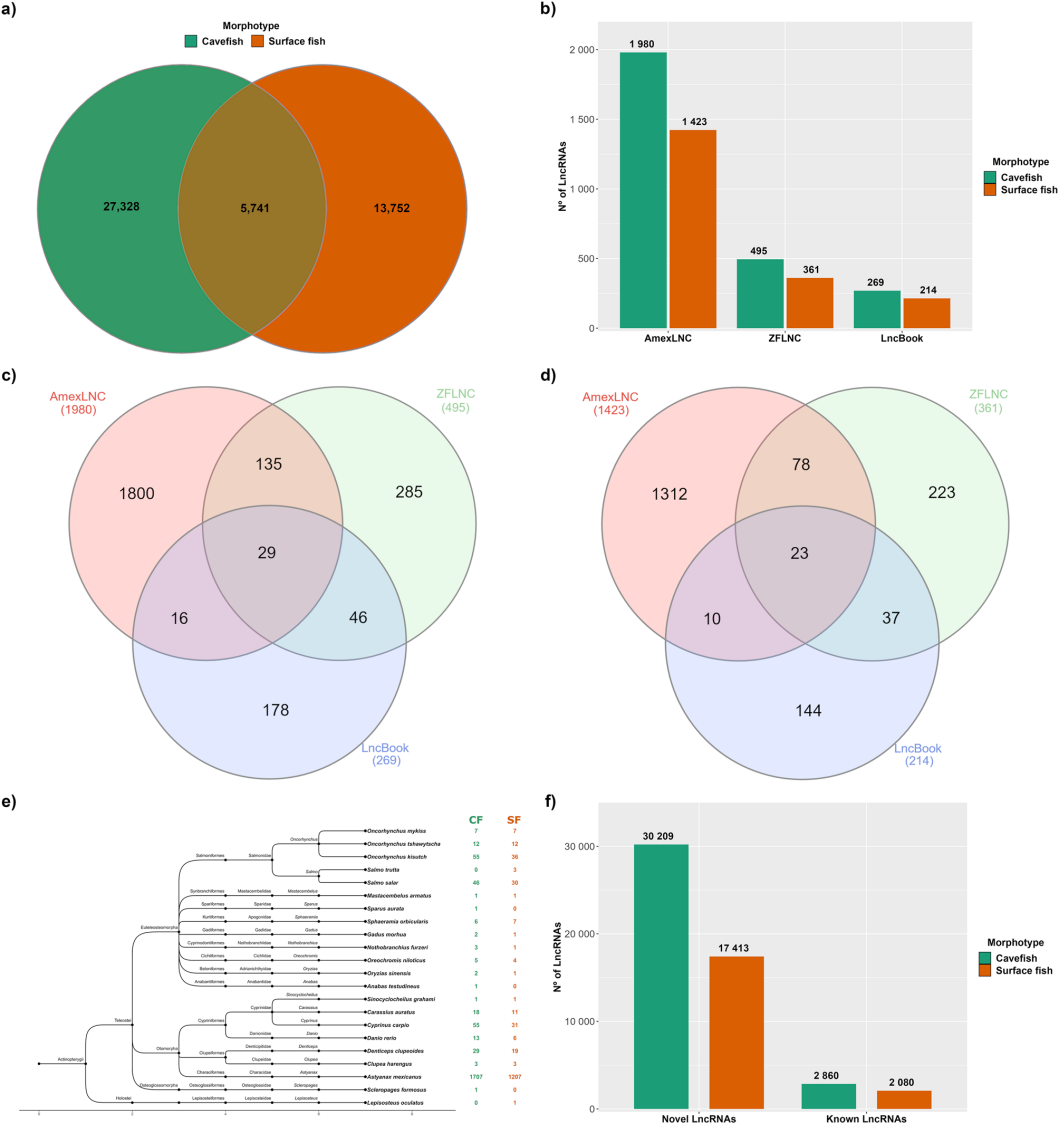
Shared lncRNAs, number of annotations against the databases and the number of novel and conserved lncRNAs. (**a**) Venn diagram showing lncRNAs that are shared between the two morphotypes; (**b**) Number of lncRNAs annotated to AmexLNC, ZFLNC and LncBook databases; Venn diagram showing lncRNAs of (**c**) cavefish and (**d**) surface fish and how the annotations were distributed across the databases. The number of lncRNAs annotated against more than one database is represented in the intersections; (**e**) Dendrogram illustrating the lncRNAs of CF and SF that were conserved among other fishes species with assemblies available in Ensembl; (**f**) Number of lncRNAs that were not annotated to any lncRNA database (Novel LncRNAs) and lncRNAs that were annotated to at least one database (Known LncRNAs), therefore, conserved lncRNAs.

From the totality of lncRNA candidates, 5,741 of them are shared between the transcriptomes of CF and SF. Thus, most of the lncRNAs are exclusive to each morphotype, i.e., 27,328 lncRNAs were only found in the CF transcriptome and 13,752 were exclusively found in the SF (Fig. 3a and Supplementary Table ST1-S5). The annotation against ESTs resulted in the validation of 941 lncRNAs from CF (Supplementary Table ST2-S6) and 526 from SF (Supplementary Table ST3-S6).

### Conserved lncRNAs

The number of lncRNAs annotated against the lncRNAs databases of *A*. *mexicanus* (AmexLNC), zebrafish (ZFLNC) and human (LncBook) is summarized in Fig. 3b. In the AmexLNC annotation step, 1980 lncRNAs of CF and 1423 of SF were annotated to at least one lncRNA (Supplementary Table ST2-S7 and ST3-S7). As for the ZFLNC step, 495 lncRNAs of CF and 361 of SF were successfully annotated (Supplementary Table ST2-S8 and ST3-S8), and, similarly, 269 and 214 lncRNAs of CF and SF, respectively, were annotated against a human lncRNA transcript (Supplementary Table ST2-S9 and ST3-S9). Following these results, most lncRNAs were uniquely annotated to a specific database, however, a few lncRNAs were annotated in more than one DB. In CF, 135 lncRNAs had a hit in both AmexLNC and ZFLNC, 46 in ZFLNC and LncBook, and 16 in AmexLNC and LncBook. Interestingly, 29 lncRNAs were annotated in all three databases (Fig. 3c). As for the SF, a similar case was found, in which 78 lncRNAs were annotated in AmexLNC and ZFLNC, 37 in both ZFLNC and LncBook, 10 in AmexLNC and LncBook, and, finally, 23 lncRNAs that were annotated in all of them (Fig. 3d).

As for the lncRNAs conservation in fishes, excluding the annotations against the *A*. *mexicanus* assembly, that, as expected, represented the most numerous annotations with 1707 hits from CF and 1207 from SF, we were able to identify multiple lncRNAs that are conserved between *A*. *mexicanus* and at least one species (Supplementary Table ST2-S10 and ST3-S10). In total, conserved lncRNAs were found in 21 other species, as represented in the dendrogram in Fig. 3e. A total of 55 lncRNAs from CF and 36 from SF matched a lncRNA from *Oncorhynchus kisutch*, and, similarly, 55 lncRNAs (CF) and 31 (SF) matched a lncRNA from *Cyprinus carpio* (Fig. 3e). Even though a considerable amount of hits were found in Salmoniformes (CF = 120; SF = 80), Cypriniformes (CF = 87; SF = 49) and Clupeiformes (CF = 32; SF = 22) no apparent relation can be identified between the number of hits and the phylogenetic proximity between *A. mexicanus* and the target species.

Furthermore, this also seems to be the case pertaining the number of hits and the number of lncRNAs and ncRNAs described for each assembly; *Salmo salar,* for instance, has 28,609 lncRNAs but only 46 hits from CF and 30 from SF, while *O. kisutch* has only 3425 sequences but was the species with most conserved lncRNAs identified (Supplementary Table ST1-S6). Additionally, some lncRNAs were conserved between multiple species, which is the case of the DN2011_c0_g1_i7 lncRNA from CF, which is conserved in *A*. *mexicanus*, *D. rerio*, *S. salar*, *O. mykiss*, *O. kisutch* and *O.* tshawytscha*.* In SF, the DN501_c29_g1_i1 lncRNA is conserved in *A*. *mexicanus*, *S. salar*, *Sphaeramia orbicularis*, *O. kisutch* and *O. tshawytscha*. The alignment details and visual representation of the top 5 lncRNAs with most species and subspecies hits, including the two previously cited, are available in Supplementary Table ST2-S10 and ST3-S10 for CF and SF, respectively.

Finally, considering the four annotation steps, we were able to identify 2860 lncRNAs of CF and 2080 of SF as known lncRNAs. In contrast, 30,209 lncRNAs of CF and 17,413 of were not annotated against any lncRNA sequence, therefore, identified as novel lncRNAs (Fig. 3f).

### LncRNAs classification

**Figure 4:**
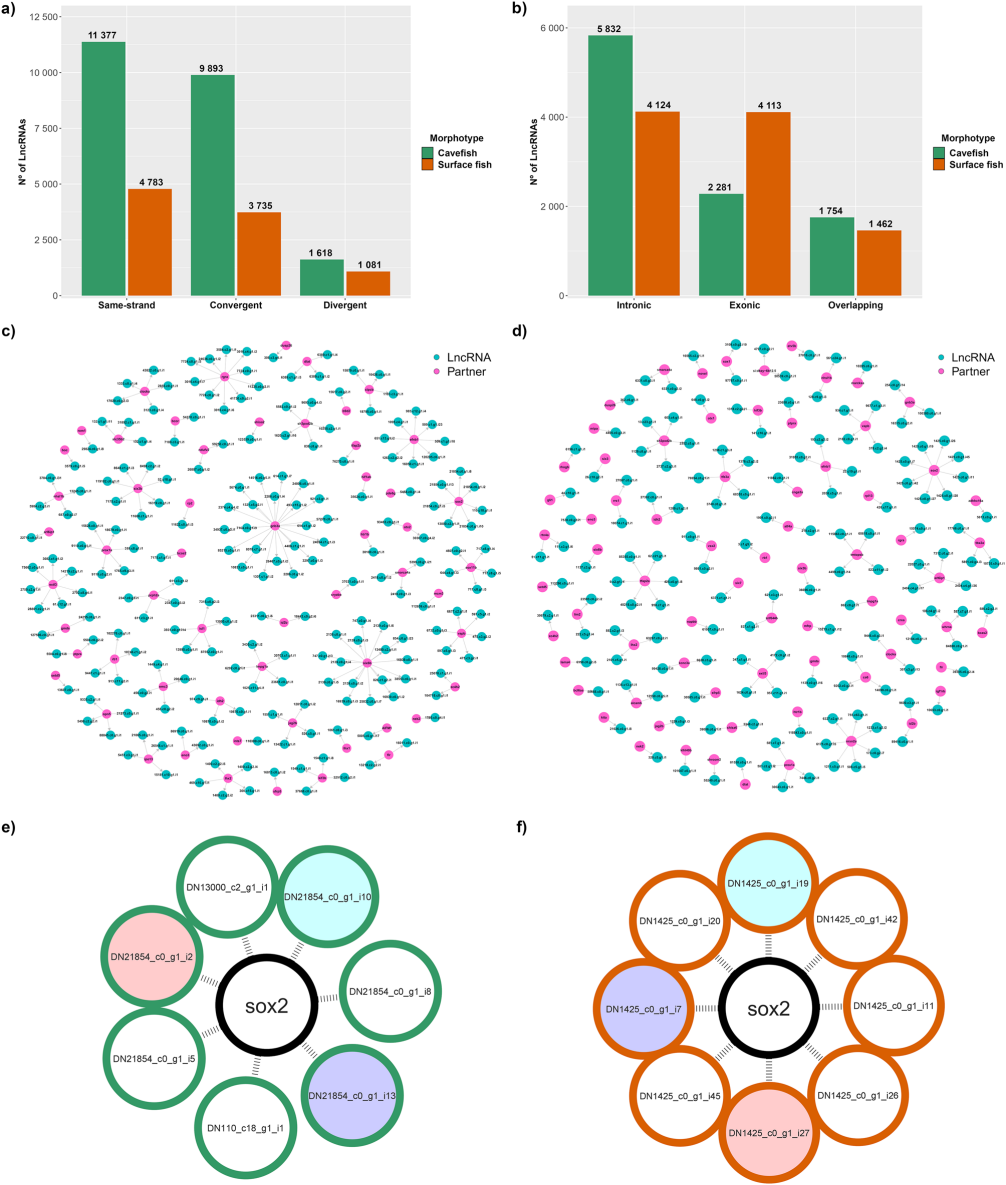
LncRNAs classification and interaction networks of lncRNAs and partners. (**a**) Distribution of intergenic lncRNAs, organized into three types: same-strand, convergent and divergent. Same-strand and convergent lincRNAs were the most abundant in both cavefish and surface fish, however, in cavefish, the number was considerably higher; (**b**) Distribution of genic lncRNAs, where cavefish had a higher number of intronic and surface fish of exonic; Interaction networks between lncRNAs of (**c**) cavefish and (**d**) surface fish and candidate genes (partners). The ‘DN’ prefix from the IDs of the lncRNAs was removed to enable better visualization. Moreover, to guarantee the legibility of these high-information figures, high-resolution versions of the interaction networks are available in Supplementary File 2 and 3, respectively; (**e**) Cavefish and (**f**) Surface fish lncRNAs, represented by outer circles, interacting with sox2 gene in the center. The tree circles with a background color other than white, represent homologous lncRNAs between morphotypes.

Intergenic lncRNAs were the most abundant in the CF transcriptome, corresponding to 22,888 (69.88%) transcripts (Supplementary Table ST2-S11). In SF, only 9599 (49.74%) lncRNAs were intergenic (Supplementary Table ST3-S11). In both cavefish and surface fish, same-strand lincRNAs were more numerous, followed by convergent and divergent (Fig. 4a). Despite the expressive difference in total numbers, the morphotypes had a similar proportion in lincRNAs. A total of 11,377 (49.71%) and 4783 (49.83%) of same-strand lincRNAs were found in CF and SF, respectively. A similar scenario was found within the convergent, in which 9893 (43.22%) were from CF and 3735 (38.91%) were from SF. Divergent represented a small part of lincRNAs, with 1618 (7.07%) and 1081 (11.26%), in CF and SF, respectively.

Genic lncRNAs displayed more differences between the two transcriptomes, with a substantial divergence in proportions (Fig. 4b). In CF, 9,867 (30.12%) genic lncRNAs were identified, of which 5832 (59.11%) were intronic, 2281 (23.12%) exonic and 1754 (17.77%) overlapping. In SF, a total of 9699 (50.26%) were observed, being 4124 (42.52%) intronic, 4113 (42.41%) exonic and 1462 (15.07%) overlapping.

### LncRNAs interactions with partners

In the CF transcriptome, we identified 32,755 (99,05%) lncRNAs interacting with 12,633 partners, while in SF, 19,298 lncRNAs were interacting with 8389 partners. Multiple lncRNAs interacting with the same partner were observed in both transcriptomes, such as the ENSAMXG00000015728, with 35 interactions, and ENSAMXG00000029878, with 46, in CF and SF, respectively. Of these interactions, we observed 205 lncRNAs interacting with 57 partners in CF (Fig. 4c and Supplementary Table ST2-S12). In SF, the number of lncRNAs was smaller, but the number of partners was higher than in CF, with 143 lncRNAs and 72 partners (Fig. 4d and Supplementary Table ST3-S12).

Among the partners, we discovered seven transcripts interacting with the *sox2* (SRY-Box Transcription Factor 2) gene in the CF, of which five of them were classified as genic sense intronic containing (intronic) and two of them, as intergenic antisense convergent lncRNAs (intergenic) (Fig. 4e and Table 2). Similarly, in the surface fish, eight transcripts were partners of *sox2*, however, all of them were intronic lncRNAs (Fig. 4f and Table 2). This classification indicates that these putative lncRNAs are transcribed in the same direction as the *sox2* and that the *sox2* is contained within them. The length varies considerably among these transcripts, ranging from 345 nt (DN1425_c0_g1_i45) to 1,189 nt (DN1425_c0_g1_i26) (Table 2).

**Table 2:** LncRNAs associated with the sox2 gene, their respective classification and morphotype, and the lncRNA length (nt).

Gene	LncRNA	Classification	Morphotype	Length
*sox2*	**DN21854_c0_g1_i13**	Intronic containing	Cavefish	530
*sox2*	DN110_c18_g1_i1	Intergenic convergent	Cavefish	1018
*sox2*	DN21854_c0_g1_i5	Intronic containing	Cavefish	709
*sox2*	DN21854_c0_g1_i8	Intronic containing	Cavefish	542
*sox2*	DN13000_c2_g1_i1	Intergenic convergent	Cavefish	1048
*sox2*	**DN21854_c0_g1_i2**	Intronic containing	Cavefish	463
*sox2*	**DN21854_c0_g1_i10**	Intronic containing	Cavefish	642
*sox2*	DN1425_c0_g1_i11	Intronic containing	Surface fish	389
*sox2*	**DN1425_c0_g1_i19**	Intronic containing	Surface fish	662
*sox2*	DN1425_c0_g1_i20	Intronic containing	Surface fish	506
*sox2*	DN1425_c0_g1_i26	Intronic containing	Surface fish	1189
*sox2*	**DN1425_c0_g1_i27**	Intronic containing	Surface fish	463
*sox2*	DN1425_c0_g1_i42	Intronic containing	Surface fish	558
*sox2*	DN1425_c0_g1_i45	Intronic containing	Surface fish	345
*sox2*	**DN1425_c0_g1_i7**	Intronic containing	Surface fish	530

LncRNAs highlighted in bold correspond to shared lncRNAs between the morphotypes.

**Figure 5:**
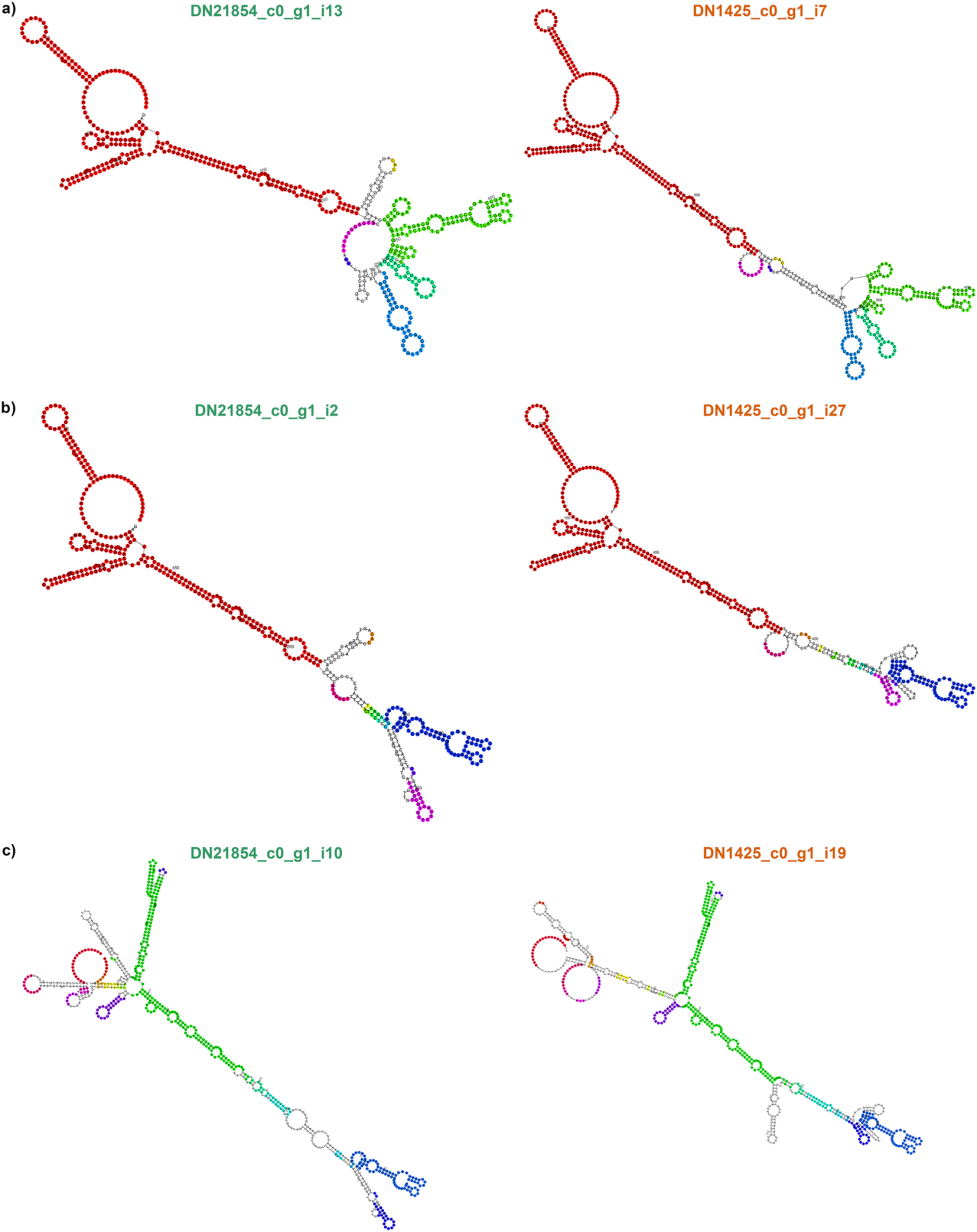
Comparison of secondary structures of RBH pairs of SOX2-OT transcripts. Motif colors represent conserved regions between the pair’s structures. (**a**) DN21854_c0_g1_i13 (CF) and DN1425_c0_g1_i7 (SF); (**b**) DN21854_c0_g1_i2 (CF) and DN1425_c0_g1_i27 (SF) and (**c**) DN21854_c0_g1_i10 (CF) and DN1425_c0_g1_i19 (SF).

According to the RBH results, CF and SF fish share three lncRNAs associated with *sox2* (Fig. 4e,f). DN21854_c0_g1_i13 (530 bp) and DN1425_c0_g1_i7 (530 bp) have a percentage of identical matches (pident) of 99.621%; DN21854_c0_g1_i2 (463 bp) and DN1425_c0_g1_i27 (463 bp) a pident of 99.566%; and DN21854_c0_g1_i10 (642 bp) and DN1425_c0_g1_i19 (662 bp) a pident of 99.533%. The secondary structures representing the solution of LCS-EPM (Longest Common Subsequence of Exact Pattern Matchings) of these SOX2-OT transcripts RBH pairs show that similarity in such transcripts is observed not only at the sequence level, but also in their structure motifs (Fig. 5a,b,c).

## Discussion

This study successfully expanded the knowledge of lncRNAs in *A*. *mexicanus*, filling a relevant gap to an important model species. From a few thousand lncRNAs described in *A*. *mexicanus,* we were able to describe tens of thousands of lncRNAs that are expressed in cave and surface fish. While some of them are shared between the morphotypes, a considerable number of them are uniquely expressed in cave or surface fish. We also identified lncRNAs conserved in several species, including human and zebrafish. Moreover, we explored their classification and interactions with other genes, creating a concise profile of lncRNAs in *A*. *mexicanus*. Some lncRNAs described here were interacting with key genes to eye development, revealing important target lncRNAs to be further studied in future research, such as the ones interacting with *sox2*.

We were successful in assembling the transcriptomes, with almost 100% of the transcripts aligning against the reference genome. Around 4–6% of the total aligned transcripts were misassemblies, and despite being a considerable percentage, it’s under the expected for the assembly method employed, since Trinity has one of the highest misassemblies rates among the de novo assemblers, as found by Yang and Smith^124^ (3.9%) and Kerkvliet et al.^125^ (17.9%). Likely, not all those transcripts represent assembly errors, since it is known that misassemblies can also be chimeric transcripts, that arise from the fusion of exons from different genes through chromosomal rearrangement or trans-splicing^124,126,127^. Therefore, it may be relevant to further explore these misassembled transcripts.

As for the coding potential, using the CP tools alone may not be sufficient to precisely identify non-coding transcripts. With our approach, using a consensus of 4 of 5 tools, we successfully evade, fully or partially, the bias effect of differences between the CP tools that could lead to a very strict classification. CP tools are, indeed, a reliable mechanism to identify non-coding transcripts, however, despite using different methods and algorithms, none of them were sufficient to precisely identify only true non-coding transcripts. Usually, CP tools use binomial classification, either a transcript is coding or non-coding, however, we were able to create a third category of transcripts classification using this method. Undetermined transcripts represent a classification where the coding potential is uncertain, for some tools they are non-coding, and for others, they are coding. This third category, alongside the EnTAP step, allowed us to annotate thousands of transcripts that seem not to be strictly non-coding transcripts. As such, we advise the use of multiple CP tools and annotation steps against a protein database to further avoid biases.

These undetermined transcripts, however, are not necessarily an issue, but rather an initial step toward the identification of potential bi-functional transcripts. Some lncRNAs have small Open Reading Frames (sORFs) that can code for small peptides with regulatory function^128–131^ and some protein-coding genes can also have non-coding isoforms^128,130,132–134^. Therefore, these undetermined transcripts may represent bi-functional transcripts and our approach may be able to discover them. However, further investigations are necessary to confirm this.

On the other hand, the divergence in lncRNAs across the chromosomes seems to not be involved with transcripts classification, but rather with differences between the reference genomes used in the mapping step. In the Pachón cave genome, for example, chromosome 1 has 133,971,750 bp^87^, while in the surface fish genome, the same chromosome has only 26,953,843 bp^45^. This difference in size is more likely to be an assembly bias, due to different sequencing and assembly methodologies, rather than a biological trait, since the number of unplaced scaffolds between the two assemblies is considerable. In the cavefish genome statistics, 170 unplaced scaffolds correspond to a total length of 29,150,210, while in the surface fish, this number is higher as 2390 scaffolds and 404,626,875 bp in length^45^. Those differences may have a role in the differences in the different proportions of genic and intergenic lncRNAs between CF and SF, as such, it should be addressed when equivalent genomes assemblies are available.

LncRNAs are known to have low primary sequence conservation in comparison to protein-coding genes^114,115,135–137^, although, conservation in short regions of the lncRNAs sequence has been observed in different species^138^. Despite that, conserved lncRNAs have been identified in vertebrates, including relatively distantly related species, such as zebrafish and humans^112,138,139^. The analysis we conducted looking for conserved lncRNAs considered the primary sequence of the lncRNAs and while the parameters could be considered strict in terms of identity and coverage for lncRNAs, it allowed us to look for sequences that were, indeed, conserved or at least had well conserved small regions. This allowed us to identify multiple lncRNAs of *A*. *mexicanus* conserved in other fishes and humans.

The interactions between lncRNAs and partners could be affected by the reference genomes, resulting in a lower number of inferred interactions than occurs. Despite that, we were able to identify interactions with almost all mapped lncRNAs and more interestingly, interactions between them with candidate genes to eye development. These interactions are very interesting, due to the regulatory role of lncRNAs, and have great potential to appoint target lncRNAs and partners, that can be used in future studies. This seems to be the case with the lncRNAs that we identified to be interacting with *sox2*. *Sox2* is a transcription factor found in the intronic region of *SOX2-OT* lncRNA (SRY-box transcription factor 2 (*SOX2*) overlapping transcript), a lncRNA that overlaps and is transcribed in the same direction as S*ox2*.

*SOX2-OT*, different from most lncRNAs, has considerable primary sequence conservation among vertebrates^140–142^. The transcription is quite complex due to the existence of multiple transcription start sites, leading to transcripts of different lengths^141,142^. These traits are very well aligned with the multiple intronic transcripts that we found interacting with *sox2*, which also have a considerable difference in length. However, further investigations are required to confirm if those transcripts are, indeed, *SOX2-OT* isoforms. If confirmed, these lncRNAs must be explored, since *SOX2-OT* plays a relevant regulatory role, acting as a miRNA sponge and upregulating or downregulating the expression of s*ox2* according to the expression tissue^140,141,143–146^. Moreover, it has been suggested that the *SOX2-OT* is involved in many processes in which s*ox2* has an important role during development, such as cell regulation, proliferation and differentiation^141,142,146^.

The *sox2* regulation may be relevant in *A*. *mexicanus*, due to *sox2* being associated with multiple processes, including maintaining the pluripotency of stem cells and the neural development and sensory organs^147–151^. Interestingly, s*ox2* has an important role in eye development, acting in different stages of retinal development and controlling the activity of the Wnt/β-catenin pathway in the retina^150,152–155^. In zebrafish, it was observed to be highly expressed in eyes and brain tissues at 28 hpf and especially expressed in the retina around 48 hpf^142^.

The role of *sox2* in *A*. *mexicanus* was explored by Ma et al.^53^ that observed the downregulation of s*ox2* through the lens of cavefishes. They suppressed the s*ox2* in the surface fishes and observed downregulation of *cryaa* expression and lens apoptosis, as such, s*ox2* seems to be involved in eye development in *A*. *mexicanus*, although, the mechanisms involved in the downregulation of the cavefish remain to be understood. In any case, the regulation of s*ox2* by *SOX2-OT* must be considered in those processes and further investigated.

While the role of protein-coding genes has been effortlessly explored in *A*. *mexicanus*, lncRNAs have been, so far, neglected and little is known about them. By constructing a concise approach to predict, identify, and describe lncRNAs, thousands of lncRNAs were found to be expressed in the eye tissue of cavefish and surface fish morphotypes of *A*. *mexicanus*. However, differences in their lncRNA profile were also observed, including lncRNAs expressed exclusively in one morphotype. Additionally, putative lncRNAs associated with relevant genes with a role in eye development were highlighted. Therefore, this work can be used as a starting point to explore lncRNAs in future studies, including those focused on differential expression, with specific targets in mind. Furthermore, we open an important precedent to the arise of studies focusing on lncRNAs expressed in *A*. *mexicanus*.

## Supplementary Information

Supplementary Information 1.

Supplementary Information 2.

Supplementary Information 3.

Supplementary Information 4.

Supplementary Information 5.

Supplementary Information 6.

## Data Availability

This Transcriptome Shotgun Assembly project has been deposited at DDBJ/EMBL/GenBank under the accession DANNHJ000000000. The version described in this paper is the first version, DANNHJ010000000. The databases, sequences, scripts and intermediate datasets generated during the current study are available in the Open Science Framework (OSF) repository, under the identifier https://doi.org/10.17605/OSF.IO/3Z7QN.
