# Supplementary material for: Discovery of putative long non-coding RNAs expressed in the eyes of Astyanax mexicanus (Actinopterygii: Characidae)
Source: Sci Rep. 2023 Jul 25;13:12051. doi: 10.1038/s41598-023-34198-5 (PMC10368750; doi:10.1038/s41598-023-34198-5)
Supplement: Supplementary file 2 — Supplementary Information 2. [file 41598_2023_34198_MOESM2_ESM.pdf]

Discovery of putative long non-coding RNAs expressed in the eyes of *Astyanax mexicanus* (Actinopterygii: Characidae)

Scientific Reports

Iuri Batista da Silva[1,2] (0000-0003-2788-5665)
David Aciole Barbosa[3] (0000-0003-3875-2307)
Karine Frehner Kavalco[2] (0000-0002-4955-2792)
Luiz R. Nunes[4] (0000-0001-9619-269X)
Rubens Pasa[2*] (0000-0002-3513-4071)
Fabiano B. Menegidio[3*] (0000-0002-4705-8352)

[1]Institute of Biological Sciences, Federal University of Minas Gerais, Belo Horizonte, 31270-901, MG, Brazil
[2]Laboratory of Ecological and Evolutionary Genetics, Institute of Biological and Health Sciences, Federal University of Viçosa Campus Rio Paranaíba, Rio Paranaíba, 38810-000, MG, Brazil
[3]Center of Biotechnology, University of Mogi das Cruzes, Mogi das Cruzes, 08780-911, SP, Brazil
[4]Center for Natural and Human Sciences, Federal University of ABC, São Bernardo do Campo, 09606-045, SP, Brazil

Corresponding authors

Fabiano B. Menegidio, Ph.D., Center of Biotechnology, University of Mogi das Cruzes (UMC), Av. Dr. Cândido X. de Almeida and Souza, 200 - Centro Cívico, Mogi das Cruzes - SP, 08780-911, Brazil - fabianomenegidio@umc.br

Rubens Pasa, Ph.D., Laboratory of Ecological and Evolutionary Genetics, Institute of Biological and Health Sciences, Federal University of Viçosa Campus Rio Paranaíba, Rio Paranaíba, 38810-000, MG, Brazil - rpasa@ufv.br

Cavefish

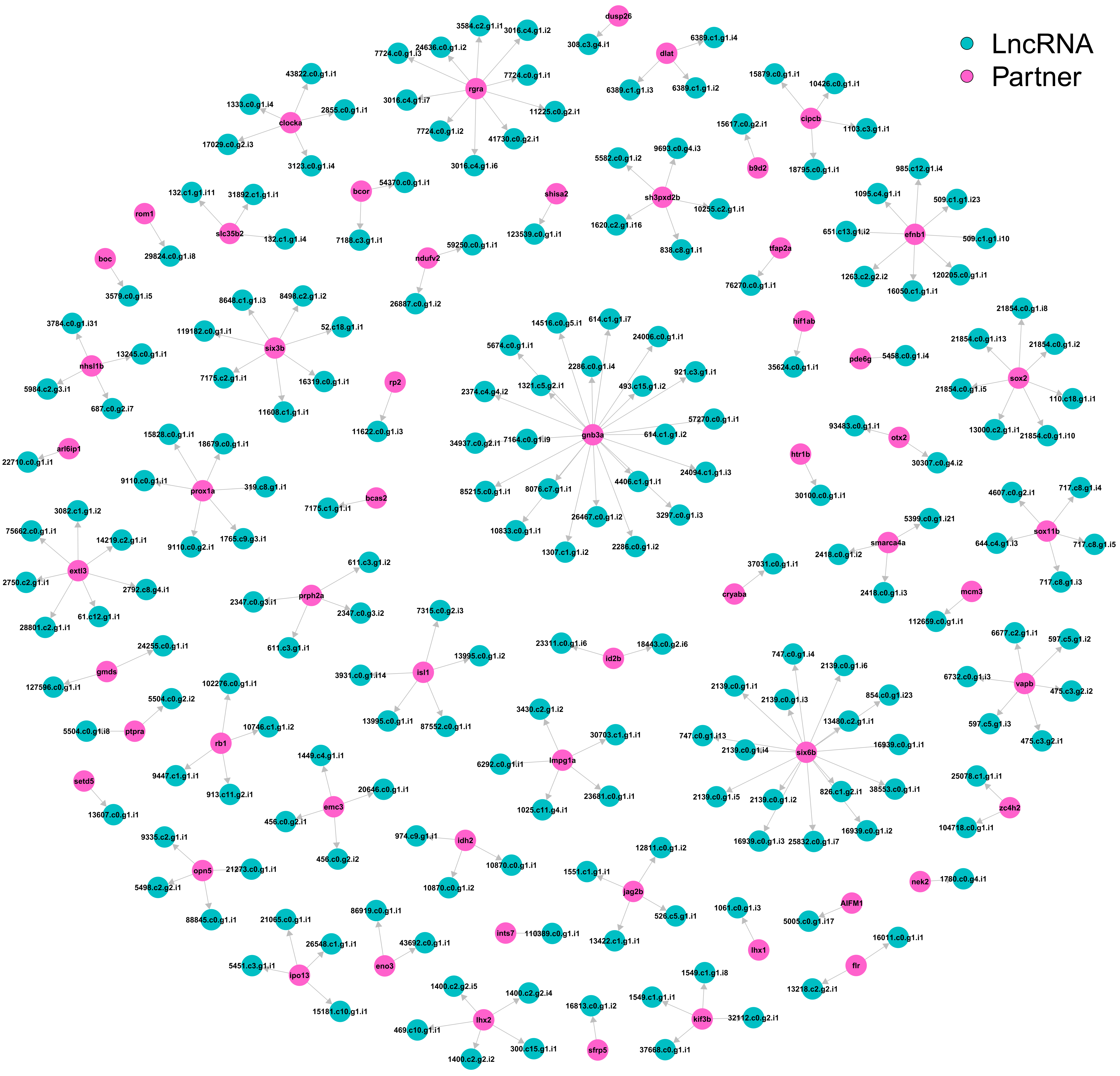

Supplementary Figure 2 Interaction networks between lncRNAs of cavefish and candidate genes (partners). LncRNAs are represented as turquoise circles and partners as pink circles. This figure is the high resolution version of **Figure 4c**. The 'DN' prefix was omitted in the lncRNAs IDs.
